# Culture Shapes Empathic Responses to Physical and Social Pain

**DOI:** 10.1037/emo0000162

**Published:** 2016-03-07

**Authors:** David Atkins, Ayse K. Uskul, Nicholas R. Cooper

**Affiliations:** 1 School of Psychology, University of Kent; 2 Department of Psychology, Centre of Brain Science, University of Essex

**Keywords:** empathy, culture, empathic accuracy, negative affect, empathic concern

## Abstract

The present research investigates the extent to which cultural background moderates empathy in response to observing someone undergoing physical or social pain. In 3 studies, we demonstrate that, East Asian and White British participants differ in both affective and cognitive components of their empathic reactions in response to someone else’s pain. Compared with East Asian participants, British participants report greater empathic concern and show lower empathic accuracy. More important, findings cannot be explained by an in-group advantage effect. Potential reasons for observed cultural differences are discussed.

As humans, our empathic abilities help us to infer the thoughts and feelings of others (Ickes, 2009) and to generate the appropriate affective and behavioral responses (Hoffman, 1987). Our ability to feel and infer others’ emotions (i.e., to empathize) is considered crucial for healthy functioning in interpersonal relationships (Blair, 2005; Eisenberg & Miller, 1987).

Research on empathic responses typically distinguishes between two components of empathy: affective and cognitive. The affective component of empathy refers to individuals’ emotional reactions in response to another person’s feelings that typically mirror the other person’s feelings or are congruent with his or her emotional state (e.g., Eisenberg & Miller, 1987; Feshbach, 1975; Hoffman, 1977). The two most commonly examined indices of affective empathy are personal distress and empathic concern (Davis, 1980, 1983b). *Personal distress* has been defined as an aversive response to witnessing someone else’s negative emotional state and is conceptualized as a self-focused emotional response associated with motivation to attenuate one’s own aversive feelings (e.g., Batson, Fultz, & Schoenrade, 1987). In contrast, *empathic concern*, synonymous to sympathy (Wispé, 1986), is usually conceptualized as an other-focused emotional response and is associated with attention turning toward the person in distress (Eisenberg et al., 1989; Schroeder, Dovidio, Sibicky, Matthews, & Allen, 1988).

The cognitive component of empathy refers to accurately recognizing another person’s thoughts and feelings (Davis, 1980; Hoffman, 1977; Ickes, Stinson, Bissonnette, & Garcia, 1990) and is mainly focused on the underlying cognitive processes such as perspective taking or accurately recognizing another’s emotions. The most commonly examined index of cognitive empathy is *empathic accuracy* that refers to individuals’ successful inferences of targets’ feelings (e.g., Kraus, Côté, & Keltner, 2010; Ma-Kellams & Blascovich, 2012) or both targets’ thoughts and feelings (Ickes, 1997, 2003).

Research on empathic responses has predominantly examined empathy as a response to observing another person’s pain or suffering. Empathic responses to others’ pain have typically been studied by investigating how individuals empathically respond when watching others being subjected to painful physical stimuli (e.g., Avenanti, Paluello, Bufalari, & Aglioti, 2005; Avenanti, Sirigu, & Aglioti, 2010; Benuzzi, Lui, Duzzi, Nichelli, & Porro, 2008), expressing painful facial expressions (e.g., Zhu, Zhang, Fan, & Han, 2007), interacting in a naturalistic social interaction (e.g., Ickes et al., 1990; Soto & Levenson, 2009), or talking about an unpleasant or sad event (e.g., Zaki, Bolger, & Ochsner, 2009). Using one of the above methods, studies have shown that the onlooker’s responses to others’ pain can be very different depending on interpersonal factors such as emotional sharing, relationship length, the interpersonal relationship between the onlooker and the target (e.g., Avenanti, Bueti, Galati, & Aglioti, 2005; Avenanti et al., 2010; Marangoni, Garcia, Ickes, & Teng, 1995; Singer, Seymour, O’Doherty, Kaube, Dolan, & Frith, 2004; Singer, Seymour, O’Doherty, Stephan, Dolan, & Frith, 2006; Stinson & Ickes, 1992) and individual difference factors such as motivation (e.g., Pickett, Gardner, & Knowles, 2004), self-monitoring (Mill, 1984), and sex (Klein & Hodges, 2001).

One potential moderator of empathic responses is cultural background. As we review below, the existing evidence on the role of culture in empathic outcomes is scarce and limited to the examination of empathic responses to social pain and certain indices of empathy only. In the present article, we extend the study of the role of culture in empathic responses by examining responses to *both* physical and social pain and assessing *both* affective and cognitive components of empathy including general negative affect as a measure of personal distress, empathic concern, and empathic accuracy among members of Western and East Asian cultural groups.

## Culture and Empathy

Accumulated evidence of cultural differences in the construal of the self and interpersonal relationships suggests that empathic responses to others’ emotional states should vary as a function of cultural background. This evidence comes predominantly from comparative studies with individuals from European American and East Asian individuals and shows that in Western cultural contexts, the self is typically experienced as an independent entity, defined primarily by its internal attributes such as preferences, desires, and traits (Kitayama, Duffy & Uchida, 2007; Markus & Kitayama, 1991). In contrast, in Eastern cultural contexts, the self is typically experienced as an interdependent and interpersonally connected entity (Kitayama et al., 2007; Markus & Kitayama, 1991), primarily defined by one’s place in social relationships and others surrounding the self. This culturally varying degree of overlap between the self and others is expected to shape individuals’ responsiveness to and level of accuracy in reading others’ pain.

There is limited empirical research conducted to examine the role of culture in empathy; two studies exist to date that are designed to investigate the affective component of empathy cross-culturally. In one observational study of preschool children across four different cultural groups (Germany, Israel, Indonesia, and Malaysia), Trommsdorff, Friedlmeier, and Mayer (2007) examined emotional responses of empathic concern and personal distress inferred from behavioral reactions to an adult experiencing a sad event (her balloon popping). They found that children from other-oriented cultural groups (Indonesia and Malaysia) displayed more personal distress than did children from individual-oriented cultural groups (Germany and Israel), whereas they did not observe any cultural group differences in empathic concern.

In another study, Cassels, Chan, and Chung (2010) examined cultural differences in empathy focusing on individual differences in empathic concern and personal distress among East Asian and European Canadian young adults. Using Davis’ (1980) *Interpersonal Reactivity Index* (IRI) to assess empathy as a trait variable, they found that Westerners reported more empathic concern, but less personal distress than did Easterners. Cassels and colleagues interpreted these findings as mirroring those by Trommsdorff and colleagues (2007) and suggested that Westerners are more other-oriented in their emotional response to another person’s distress than Easterners. Thus, these two studies show diverging patterns of emotional responses between cultural groups, with Westerners reporting greater empathic concern than Easterners, and Easterners reporting greater personal distress than Westerners in response to others’ negative experiences.

Two recent studies designed to examine empathic accuracy as an index of cognitive empathy have reported mixed findings regarding the role of cultural background. Soto and Levenson (2009) asked participants from four cultural groups (African American, Asian American, European American, and Mexican American) to observe videos of four unknown dyads, each from one of the same four cultural groups, discuss a relational issue and to infer the emotions of one of the pair. The researchers measured empathic accuracy of emotional intensity and valence (positive and negative) dynamically over time as participants watched videos. They found no cultural differences in empathic accuracy.

In another line of research, Ma-Kellams and Blascovich (2012) studied cultural differences in empathic accuracy as a function of target familiarity (stranger vs. close other). They asked European American and East Asian participants to infer the emotions of both strangers and close others describing a recent emotional experience, and assessed participants’ empathic accuracy of emotional intensity for specific emotions (see also Côté et al., 2011; Kraus, Côté, & Keltner, 2010). In line with past research showing that Easterners tend to be more concerned with the feelings of others with whom they share a relational link (e.g., Cousins, 1989; Heine, 2001; Kanagawa, Cross, & Markus, 2001), Ma-Kellams and Blascovich found that East Asians inferred the emotions of close others more accurately than did European Americans. Ma-Kellams and Blascovich also demonstrated that European American participants inferred the emotions of strangers more accurately than did East Asian participants. This finding is in line with other research demonstrating that compared with Westerners, Easterners tend to be less concerned with the feelings of individuals with whom they have no relational link (Chen, DeSouza, Chen, & Wang, 2006; Chen, Hastings, Rubin, Chen, Cen, & Stewart, 1998; Yuki, Maddux, Brewer, & Takemura, 2005), Thus, overall, findings concerning cultural differences in cognitive empathy lack consistency across the limited number of existing studies.

## The Present Research

To date the existing culture comparative studies on empathy that we reviewed above examined exclusively either affective or cognitive components of empathy in response to social (not physical) pain. We asked whether empathic responses to perception of painful stimuli are moderated by cultural background with a goal to contribute to the limited pool of studies on culture and empathy with further evidence in this area and thus expanding the field by focusing on both physical pain and social pain, and measuring both affective and cognitive components of empathy.

In the first two studies reported below, we tested the following predictions that are inspired by existing research on the cultural variations of the self and interpersonal relationships, as well as research on cultural variations in components of empathy reviewed above. First, we predicted that individuals of East Asian background, relative to individuals of White British background, would be more likely to suppress the expression of affective empathic responses of personal distress *and* empathic concern in response to others’ negative emotional states. This prediction is based on the literature demonstrating that one way members of East Asian cultures maintain interpersonal harmony is by monitoring the expression of their emotions that may consequently disrupt otherwise harmonious relationships (Bond & Hwang, 1986; Chiu & Kosinski, 1994; Markus & Kitayama, 1994). For example, East Asians exhibit a more positive association between emotional suppression and interpersonal harmony (Wei, Su, Carrera, Lin, & Yi, 2013) and a tendency to suppress both positive and negative emotions to maintain interpersonal harmony (Chiang, 2012). In fact, East Asian individuals generally have the propensity to display emotions less in comparison to their European American counterparts (e.g., Ekman & Friesen, 1969; Matsumoto, 1990; Matsumoto, Takeuchi, Andayani, Kouznetsova, & Krupp, 1998). Studies have shown that Americans, compared with Japanese, report feeling emotions more intensely and for a longer duration (Matsumoto, Kudoh, Scherer, & Wallbott, 1988; Mesquita & Karasawa, 2002) and are less likely to mask emotions, closing the gap between internal emotional states and outward expression (Gross & John, 1995). In fact, emotional suppression is associated with greater levels of depression and reduced levels of life satisfaction for European Americans, whereas the same association is not evident among Hong Kong Chinese (Soto, Perez, Kim, Lee, & Minnick, 2011). Thus, the expression of affective empathic responses of personal distress and empathic concern among individuals of White British background might be important in regulating psychological functioning.

Second, we predicted that individuals of East Asian background would exhibit greater empathic accuracy than would individuals of White British background. This prediction is based on previous studies demonstrating that, compared with European Americans, East Asians tend to pay greater attention to others’ needs, desires, and goals (e.g., Yamagishi, 1988) and have their own feelings, thoughts, and needs closely linked to others’ feelings, thoughts, and needs (e.g., Kitayama, Markus, & Kurokawa, 2000; Mesquita & Karasawa, 2002; Uchida, Norasakkunkit, & Kitayama, 2004). This prediction also fits past findings showing that East Asians relate to others by following cultural expectation of behaving in ways that align with others’ emotional states, thereby fulfilling the goal to maintain interpersonal harmony; a goal of greater importance among Easterners compared with Westerners (e.g., Ohbuchi, Fukushima, & Tedeschi, 1999). Thus, Easterners may exhibit higher empathic accuracy than Westerners because a more accurate understanding of another’s emotional state would assist behavior in ways that maintain interpersonal harmony.

To test these predictions, in Study 1, we asked participants to observe a physically painful situation and assessed self-reported affect ratings as an index of personal distress. In Study 2, we asked participants to observe socially painful situations and examined self-reported affect ratings in response to these situations, as well as empathic accuracy and feelings of empathic concern.

## Study 1

In Study 1, we examined cultural differences in how individuals emotionally respond to seeing another person experiencing physical pain. The experimental stimuli consisted of four videos depicting a hand being punctured by a needle and three control conditions, similar to the visual stimuli used in previous research investigating empathy for pain (e.g., Avenanti et al., 2005; Minio-Paluello, Baron-Cohen, Avenanti, Walsh, & Aglioti, 2009; Valeriani et al., 2008). British and East Asian participants reported their affective state while watching the videos, as an indicator of personal distress.

### Method and Design

#### Participants

Thirty-eight participants who self-identified as British (22 women, *M*_age_ = 20.53 years) and 33 participants of East Asian origin (25 women, *M*_age_ = 23.70 years) studying at a British university participated in a study on interpersonal relationships in exchange for £3. The East Asian sample consisted of 19 Chinese, 4 Japanese, 4 Taiwanese, 4 Vietnamese, 2 Bruneians, 2 Koreans, and 1 Malaysian, 15.6% of whom reported having resided in the United Kingdom for less than 6 months, 34.4% for up to a year, 12.5% between 1 and 2 years, 28.1% between 2 and 5 years, and 9.4% between 5 and 10 years. East Asian participants (*M* = 23.79, *SD* = 2.98) were significantly older than British participants (*M* = 20.53, *SD* = 5.53), *t*(69) = 2.94, *p* = .004, *d* = 1.44. On a 5-point scale (1 = *not fluent at all* to 5 = *very fluent*), East Asian participants self-rated that they were *average* to *somewhat fluent* in English (*M* = 3.67, *SD* = .69). Preliminary analyses showed that all analyses conducted in this study remained unchanged when age was controlled for; hence age is not considered further.

#### Procedure and materials

Participants completed the study individually in the lab. Initially, participants completed an online questionnaire containing demographic questions. Participants then observed four approximately 10-s long videos in random order. The experimental condition (pain condition) showed a needle puncturing a female White hand (target) at a 45**°** angle. Three standard control conditions that are commonly used in the literature (see Avenanti et al., 2005; Minio-Paluello et al., 2009; Valeriani et al., 2008) were generated where: (a) the needle was replaced by a Q-tip; (b) the hand was replaced by a tomato; and (c) the hand and the needle were replaced by a tomato and Q-tip, respectively. As participants observed videos, they were instructed to provide a continuous report of their personal affective state using a rating dial. Following each video, participants were asked to indicate how much pain they thought the target was feeling using a perceived pain measure. At the end of the experiment, participants were thanked, debriefed, and paid.

##### Affect rating

Participants were instructed to provide a continuous report of their positive and negative affective state as they watched each video by using a rating dial. The rating dial used to measure participants’ affective state was connected to the computer via a USB port (similar to Levenson & Ruef, 1992) and manipulated a 9-point scale (1 = *very negative* to 9 = *very positive*) on the screen. The rating dial scale position was set to the midpoint (neutral) at the start of each video presentation and was designed to capture the participant’s affect rating every 0.5 s.

##### Perceived pain

The perceived pain measure was used to assess participants’ perception of the target pain for each video condition. The measure served to check the validity of the pain condition (i.e., that the pain condition was perceived as more painful than the control conditions) and to examine whether members of the two cultural groups perceived comparable levels of pain in the target. Participants indicated their responses on a 6-point scale (1 = *no hurt* to 6 = *hurts worst*) with each point accompanied by a cartoon face progressively appearing more distressed as the values on the scale increased.

### Results

First, we examined the cultural differences in perceived pain to check the validity of the pain condition, and to determine whether the two cultural groups perceived comparable levels of pain in the pain condition. Next, to test the moderating role of culture in emotional empathy in response to observing physical pain, we examined the cultural differences in affect rating. We conducted separate 2 × 4 repeated-measures analysis of variance (ANOVA), with cultural group (British vs. East Asian) as the between-subjects variable, and condition (needle-hand; needle-tomato; Q-tip-hand; Q-tip-tomato) as the within-subjects variable for perceived pain and affect rating as dependent variables (see Table 1 for descriptive statistics). We report any effects of sex in a footnote.[Table-anchor tbl1]

#### Perceived pain

The ANOVA with perceived pain as the dependent variable revealed a significant main effect of condition, *F*(3, 207) = 125.30, *p* < .001, η_p_^2^ = .65. Participants perceived significantly greater target pain in the pain condition (i.e., needle puncturing a hand) compared with all the control conditions (all *p*s < .001, range of *d*′s = 1.00–2.70) demonstrating that the experimental manipulation worked as expected. Moreover, participants perceived significantly more pain in the needle-tomato condition compared with control conditions containing the Q-tip (all *p*s < .001, range of *d*s = .99–1.26). The main effect of cultural group, *F*(1, 69) = .18, *p* = .67, *d* = .66, and the cultural Group × Condition interaction were not significant, *F*(3, 207) = .40, *p* = .751, η_p_^2^ = .01, indicating that each cultural group perceived comparable levels of target pain in all conditions. This finding suggests that any observed cultural differences in affect rating are not likely to be attributed to cultural differences in perceived target pain.^1^

#### Affect rating

To compute participants’ own affect in response to videos, we first identified the time window from the onset of pain (i.e., when the needle/Q-tip touches the hand/tomato) to the end of the presentation, which lasted for 7 s and contained 15 affect rating scores. Next, we computed mean affect rating scores for each video using these scores.

The ANOVA with affect rating as the dependent variable revealed a significant main effect of condition, *F*(3, 207) = 64.27, *p* < .001, η_p_^2^ = .48. Participants reported significantly more negative affect in the pain condition compared with all control conditions (all *p*s < .001, range of *d*s = .68–1.86). In addition, participants reported significantly more negative affect in the needle-tomato control condition compared with control conditions containing Q-tips (all *p*s < .001, range of *d*s = .68–1.23). Finally, participants reported significantly more negative affect in the Q-tip-hand condition compared to the Q-tip-tomato condition (*p* = .005, *d* = .30).

This analysis also revealed a significant main effect of cultural group, *F*(1, 69) = 7.81, *p* = .007. British participants reported more negative affect overall compared with East Asian participants, *d* = .67. These two main effects were qualified by a cultural Group × Condition interaction, *F*(3, 207) = 4.69, *p* = .003, η_p_^2^ = .06. The simple main effects analysis conducted to decompose this interaction showed that British participants reported significantly more negative affect when observing physical pain compared to East Asian participants, *F*(1, 69) = 12.10, *p* = .001, *d* = .83, whereas the two cultural groups did not differ significantly from each other in any of the other control conditions (all *p*s > .09).^2^

### Discussion

This study demonstrated cultural group differences in affect rating when observing a person undergoing physical pain using a commonly used procedure for studying empathic responses (e.g., Avenanti et al., 2005; Minio-Paluello et al., 2009; Valeriani et al., 2008). Specifically, British participants reported more negative affect than did East Asian participants when watching a needle puncturing a hand, even though levels of perceived target pain were comparable across the two cultural groups. The two groups did not differ in their affect ratings when watching the control videos that did not depict physical pain. The findings are in line with past research that demonstrates less intense levels of emotional experience among Easterners compared with Westerners (Chiang, 2012; Wei et al., 2013), but do not follow findings that demonstrate greater personal distress among Easterners compared with Westerners (i.e., Cassels et al., 2010; Trommsdorff et al., 2007).

Thus, here we provide initial, and novel, evidence for cross-cultural differences in affective empathic responses (i.e., affect rating) to physical pain. However, it remains to be seen whether the observed cultural group difference would extend to situations where individuals witness others experiencing *social* pain. Moreover, in this initial study we used affect rating as an index of empathy, and we, therefore, do not know whether an examination of other indices of empathy would reveal a similar pattern of cultural group differences. To address these questions, in Study 2 we examined empathic responses to social pain using other common indicators of affective and cognitive empathy: empathic concern and empathic accuracy.

## Study 2

Social pain is defined as an emotional reaction to the social exclusion or devaluation of any relationships that are valued (MacDonald & Leary, 2005). Thus, empathizing with social pain can be defined as an affective, or cognitive, reaction to another person’s emotional reaction as that person responds to social pain. Of interest to the authors, social pain may share many of the neurobiological and neural mechanisms that underlie physical pain (Eisenberger, 2012; Eisenberger & Lieberman, 2004; Eisenberger, Lieberman, & Williams, 2003; but also see Woo et al., 2014). Furthermore, MacDonald and Leary (2005) posit the similarities shared between physical and social pain with regard to their relationships to other psychological constructs, such as introversion-extraversion, social support, anxiety-fear, depression, and defensive aggression. There are, however, also notable differences between the two types of pain. For example, reliving and re-experiencing social pain is easier, more intense and detrimental to cognitively demanding tasks in comparison to physical pain (Chen, Williams, Fitness, & Newton, 2008). Thus, the pattern of findings observed in Study 1 in relation to empathic responses to observing another person suffering from physical pain may or may not generalize to empathic responses to observing another person suffering from social pain. Therefore, we conducted Study 2, with a goal to examine cultural differences in empathic responses in the context of social pain.

The experimental stimuli in this study consisted of videos of British individuals (whom we call targets from now on) describing negative social experiences they experienced in the past. A group of British and East Asian participants watched these videos and reported (a) their own affective state while watching the videos (as in Study 1), (b) their empathic concern for the target in the video, (c) inferences of the target’s emotional state, and (d) the perceived levels of pain (as in Study 1).

### Method

#### Participants

Forty-five participants self-identified as British (22 women, *M*_age_ = 22.56 years) and 41 participants of East-Asian origin (32 women, *M*_age_ = 24.49 years) studying at a British university participated in a study on interpersonal relationships in exchange for £5. The East Asian sample consisted of 29 Chinese, 2 Japanese, 3 Taiwanese, 1 Vietnamese, 2 Bruneians, 1 Korean, 1 Malaysian, 1 Singaporean, and 1 Filipino, 53.7% of whom reported having resided in the United Kingdom for less than 6 months, 4.9% for up to a year, 9.8% between 1 and 2 years, 14.6% between 2 and 5 years, 9.8% between 5 and 10 years, and 7.3% for more than 10 years. Using the same scale as in Study 1, on average, East Asian participants self-rated that they were *average* to *somewhat fluent* in English (*M* = 3.49, *SD* = .78). The two samples were comparable in age, *t*(84) = .85, *p* = .40; thus, age was not explored any further.

#### Stimulus development

To create the social pain stimuli, we conducted a prestudy following a similar protocol to that used by other researchers (e.g., Ma-Kellams & Blascovich, 2012; Zaki, Bolger, & Ochsner, 2008). Eight female British individuals were invited to the lab to be videotaped while describing two socially negative events they experienced in the past. They received £4 for this task. As with Soto and Levenson (2009), female targets were used because women have the tendency to express more sadness to negative events (Hess, Senécal, Kirouac, Herrera, Philippot, & Kleck, 2000), are more emotionally expressive than men (Hall, Carter, & Horgan, 2000; Gross & John, 1995; LaFrance & Banaji, 1992), and stimulate greater empathic accuracy than men (Klein & Hodges, 2001; Levenson & Ruef, 1992). Before recording each event, to aid the recall experience, targets were asked to give each event a title and write about the relevant background. Targets were then recorded as they were talking about each negative event. Following the completion of the recording, targets rated the intensity (1 = *not intense at all* to 9 = *extremely intense*) and affective valence (1 = *extremely negative* to 9 = *extremely positive*) of the actual recall experience, which was later used for video selection for the main study. In addition, targets completed the original Positive and Negative Affect Schedule (PANAS; Watson, Clark, & Tellegen, 1988) immediately after each recording using a 5-point Likert scale (1 = *very slightly* or *not at all* to 5 = *extremely*) to reflect their feelings when they described their experiences. Video targets granted permission to the researchers to use the recorded videos for future research.

The following criteria were used to select the videos used in the current study. The most intense videos were first short-listed on the basis of affect valence (less than 3 on the affect valence 9-point scale) and intensity ratings (greater than 7 on the intensity 9-point scale), which resulted in six videos from a total of 16 videos. The final two videos were selected by the principal researchers from this short-list on the basis of video content and ease of comprehension.^3^ Videos with easily comprehensible English speakers (e.g., who used no slang or idioms and had clear, articulate speech) and content describing experiences likely to be common to all participants regardless of cultural background (i.e., being a victim of bullying, a relationship break-up) were selected.

#### Main study procedure and measures

As in Study 1, participants completed the study individually in the lab and were initially presented an online questionnaire containing demographic questions. Next, participants watched the two social pain videos in their entirety while continuously indicating their own affective state in response to the videos using the affect rating dial described in Study 1. Following each video, participants indicated how much pain they thought the target was feeling while describing the event using the perceived pain measure. Participants then completed the same PANAS items completed by targets in the stimulus development phase, with instructions to judge the *target’s* feelings as the target was recalling the event in the video. Finally, participants indicated their feelings of empathic concern they experienced while watching the videos using a subset of items from the *Emotional Response Questionnaire* (ERQ; Coke, Batson, & McDavis, 1978). Once participants had watched all videos and indicated their responses on all measures, they were thanked, debriefed and paid for their participation.

#### Perceived pain

Participants’ perception of target’s pain was measured as in Study 1 and served as a manipulation check to assess whether members of the two cultural groups perceived comparable levels of pain in the target.

#### Affect rating

As in Study 1, affect rating was measured continuously during each video presentation using a rating dial (see Study 1 for details).

#### Empathic concern

A subset of items from the ERQ (Coke, Batson, & McDavis, 1978), a commonly used scale of empathic concern (e.g., Batson, Fultz, & Schoenrade, 1987) that consists of six emotional adjectives (*compassionate*, *sympathetic*, *moved*, *tender*, *warm*, and *soft-hearted*), was used to measure participants’ feelings of empathic concern (target_1_: α_BR_ = .88, α_EA_ = .74; target_2_: α_BR_ = .80, α_EA_ = .58). Each emotional adjective was rated on a 5-point Likert scale (1 = *very slightly or not at all* to 5 = *extremely*).

#### Empathic accuracy

To compute empathic accuracy scores, we used a similar procedure to that used by Côté et al. (2011) (see also Kraus, Côté, & Keltner, 2010; Ma-Kellams & Blascovich, 2012). Absolute difference scores between each PANAS emotion score reported by the targets in the videos and those reported by the participants were calculated. For both targets, all emotions were collapsed to produce an empathic accuracy score (target_*1*_*:* α_BR_ = .82, α_EA_ = .90; target_2_: α_BR_ = .87, α_EA_ = .76) for each target. To ease interpretation, the average score was multiplied by −1 so that a lower score reflected lower empathic accuracy and a higher score reflected higher empathic accuracy.

### Data Preparation and Analysis

In the present study, participants were presented with stimuli in which the video content were considerably longer and therefore contained more emotion inducing cues, compared with the stimuli presented in Study 1. In addition, although video targets were instructed to describe socially negative events they experienced in the past, given the naturalistic quality of the stimuli videos, on occasion targets also described more positive aspects of the event that could potentially yield positive affective responses from participants (e.g., becoming attached to others before having to say goodbye). Consequently, participants’ responses varied widely both in emotional intensity and emotional valence across the time series. Thus, computing a mean summary of affective responses across the whole time series, as we did in Study 1, would not be suitable given the wide within participant variation in affective responses. To capture the rich nature of the emotional content of the videos, we used analytical techniques that would allow us to use the data in their entirety in each video time series and to examine participants’ negative and, although not our primary focus, explore positive affective responses.

For each video, we recorded participants’ affective responses every .5 s in real-time; therefore, yielding three types of responses at any one time: Participants could rotate the dial clockwise (indicating a positive affective reaction to the video at that specific time), counterclockwise (indicating a negative affective reaction to the video at that specific time), or not move the dial at all (indicating no affective reaction to the video at that specific time). To compute participants’ affective responses to videos, we first identified and summed the total number of positive and negative affective reactions (i.e., the number of times a participant indicated an affective state change on the rating dial), and the total number of “no affective reactions” in each time series (i.e., the number of times a participant did not manipulate the rating dial). Using these data, we calculated the proportion of affective reactions, separately for negative affect, positive affect, and no affective reactions, against each participant’s total number of affective reactions in the time series. As proportions range between 0 and 1 and this constitutes compositional data, to enable multivariate testing we followed guidelines concerning the handling of compositional data by Pennington, James, McNally, Pay, and McConachie (2009) who suggest taking the logarithm of the ratio between the proportion of interest and a reference proportion. We initially added 1 to each of the affective proportions (positive affect, negative affect, and no affective reactions) to create adjusted affective proportions; this computation enables the computation of logarithms. We computed logarithms on the ratio between the adjusted negative affect proportion and the adjusted no affective reaction proportion (i.e., reference). The same computation was then calculated with the adjusted positive affect proportion as the numerator. These transformed variables known as “log-ratios” enable the use of multivariate analytical techniques.

Analyzing the components of the affect rating time series using the approach explained above has two benefits. First, examining affective reactions across the whole time series, as opposed to sampling data from time windows coded for negative or positive content allows the examination of each participant’s idiosyncratic response to each target. Second, by examining affective *reactions* across the time series we are able to examine both negative and positive affective reactions separately as opposed to a single mean score of affect.

### Results

We first examined whether the two cultural groups perceived comparable levels of pain in video targets. We then examined cultural differences in the outcome measures (affect rating, empathic concern, and empathic accuracy) in response to observing the social pain videos. Unless indicated otherwise, we conducted a series of 2 × 2 repeated measures ANOVA with each outcome measure as dependent variables, cultural group (British vs. East Asian) as the between-subjects variable and video target (video target_1_ vs. video target_2_) as the within-subjects variable (see Table 2 for descriptive statistics). As before, we report any effects of sex in a footnote.[Table-anchor tbl2]

#### Perceived pain

The analysis revealed no significant main effect of cultural group, *F*(1, 84) = .12, *p* = .73, demonstrating that cultural groups perceived comparable levels of pain. However, we found a significant main effect of video target, *F*(1, 84) = 4.05, *p* = .05, with more pain perceived in video target_1_ (*M* = 4.72, *SD* = .78) compared to video target_2_ (*M* = 4.50, *SD* = .78). There was no significant Cultural group × video Target interaction, *F*(1, 84) = .43, *p* = .51, suggesting that cultural groups perceived comparable levels of pain within each target. We added video target as an additional factor in the analyses below as the two stimuli videos differed significantly in perceived pain. Note, however, that further analyses showed that the findings reported below remained significant when mean scores for each outcome variable were collapsed across the two video targets.

#### Affect rating

We present the proportional affect rating scores in Table 2 as these scores are easier to interpret compared to the logged-ratios. We subjected the logged ratios of adjusted proportional affect rating scores (positive affect and negative affect) to the adjusted proportional no affect rating scores to a 2 × 2 × 2 repeated-measures ANOVA with cultural group (British vs. East Asian) as the between-subjects variable and both affective valence (positive vs. negative) and video target (video target_1_ vs. video target_2_) as the within-subjects variables. This analysis revealed a significant main effect of cultural group, *F*(1, 84) = 7.54, *p* = .01, η_p_^2^ = .08, demonstrating that the British cultural group reported greater proportions of positive and negative affect across both video targets (British: *M* = −3.66, *SD* = .40; East Asian: *M* = −3.90, *SD* = .39), *d* = .61. In addition, there was a significant main effect of affective valence, *F*(1, 84) = 163.57, *p* = .001, η_p_^2^ = .66; overall, a greater proportion of negative affect (*M* = −3.66, *SD* = .39) was reported across both video targets compared with the proportion of positive affect (*M* = −3.89, *SD* = .44), *d* = .55. There was also a significant main effect of video target, *F*(1, 84) = 22.44, *p* = .001, η_p_^2^ = .21; video target_2_ (*M* = −3.67, *SD* = .47) elicited greater proportions of affect compared with video target_1_ (*M* = −3.88, *SD* = .44), *d* = .46. The video Target × Affective valence interaction was also significant, *F*(1, 84) = 133.09, *p* = .001, η_p_^2^ = .61. The simple main effects revealed video target differences in proportional negative affect, *F*(1, 84) = 66.11, *p* = .001, η_p_^2^ = .44, showing greater proportional negative affect was elicit in response to video target_2_ (*M* = −3.49, *SD* = .45) compared with video target_1_ (*M* = −3.84, *SD* = .43), *d* = .79. There was no significant difference in proportional positive affect as a function of video target, *F*(1, 84) = 2.18, *p* = .14, η_p_^2^ = .03. In addition, there was neither a significant cultural Group × Video target, *F*(1, 84) = .28, *p* = .60, η_p_^2^ = .003, nor a significant cultural Group × Affective valence interaction, *F*(1, 84) = .17, *p* = .68, η_p_^2^ = .002. Finally, the three-way interaction was also not significant, *F*(1, 84) = .01, *p* = .91, η_p_^2^ = .001.^4^

#### Empathic concern

There was a significant main effect of cultural group, *F*(1, 84) = 10.62, *p* = .002, with British participants reporting more overall empathic concern (*M* = 1.85, *SD* = .99) compared with East Asian participants (*M* = 1.26, *SD* = .65), *d* = .70. However, there was no significant main effect of video target, *F*(1, 84) = 1.11, *p* = .30. The cultural Group × Video target interaction was marginally significant, *F*(1, 84) = 3.80, *p* = .06, η_p_^2^ = .04. Unpacking the interaction revealed a significant cultural difference in empathic concern for video target_2_, *F*(1, 84) = 17.53, *p* = .001, with British participants reporting more empathic concern (*M* = 1.89, *SD* = 1.01) compared with East Asian participants (*M* = 1.12, *SD* = .64), *d* = .91, in response to this target. The cultural difference in empathic concern scores in response to video target_1_’s social pain was marginally significant, *F*(1, 84) = 3.55, *p* = .06, again British participants reported more empathic concern (*M* = 1.81, *SD* = 1.16) compared with East Asian participants (*M* = 1.39, *SD* = .86), *d* = .41.^5^

#### Empathic accuracy

There was a significant main effect of cultural group, *F*(1, 84) = 4.16, *p* = .04; East Asian participants were significantly more empathically accurate (*M* = −1.10, *SD* = .32) compared with British participants (*M* = −1.23, *SD* = .39), *d* = .36. There was also a significant main effect of video target, *F*(1, 84) = 6.99, *p* = .01, with more empathic accuracy shown in response to video target_2_ (*M* = −1.23, *SD* = .37) compared with video target_1_ (*M* = −1.11, *SD* = .33), *d* = .34. However, the cultural Group × Video target interaction was not significant, *F*(1, 84) = .23, *p* = .63. Although it was easier to infer emotions in video target_2_ the cultural difference in empathic accuracy scores remained.^6^

### Discussion

This study extends the findings reported in Study 1 to social pain stimuli and replicates the pattern of cultural differences observed in response to observing physical pain stimuli. Specifically, the findings revealed that British participants reported a greater proportion of affective reactions (regardless of valence) compared to East Asian participants in response to others’ social pain. These cultural differences in proportional affective reactions were evident even though members of both cultural groups perceived the same levels of pain in the video target, and remained when we repeated the analysis controlling for perceived pain. British participants also reported more empathic concern for the targets experiencing social pain than did East Asian participants. However, East Asian participants were more empathically accurate than British participants. Thus overall, this study demonstrates that there is cultural variation in both affective and cognitive components of empathy. The observed cultural group differences in empathic concern (but not in affect rating) replicate Cassels et al.’s (2010) finding (Westerners exhibiting higher trait level empathic concern compared with Easterners).

One design feature of this study was that the targets whose social pain stories that our participants watched were of White British origin who told their stories in their native language (English). This raises in-group advantage effect as a potential explanation for the observed findings. In-group advantage effect in the context of empathy suggests that an observer may experience greater empathy for individuals perceived as in-group members compared with individuals perceived as out-group members. Such an explanation would be in line with past evidence demonstrating that individuals recognize emotions of members of their own cultural group more accurately compared to nonmembers (for relevant meta-analyses see Elfenbein & Ambady, 2002a, 2002b) and Preston and de Waal’s (2002) Perception Action Model who highlight the importance of the similarity between observer and target in the activation of empathic emotions. Although in-group advantage would not help explain the currently observed cultural differences in empathic accuracy (i.e., East Asian participants were more empathically accurate compared with British participants despite the fact that targets were of White British origin), we wanted to rule out this possibility in the next study. In Study 3, we assessed the same empathic outcomes reported in Study 2 with participants of British and Chinese origin as they observed British *and* Chinese targets, speaking English and Cantonese, respectively, to examine whether an in-group advantage effect is a likely account that underlies the cultural differences in empathic responses observed so far.

## Study 3

To examine whether an in-group advantage effect explains the cultural differences observed in empathic responses reported so far, we asked a group of British and Chinese participants to report (a) their own affective state while watching the videos (as in Studies 1 and 2), (b) their empathic concern for the target in the video (as in Study 2), (c) inferences of the target’s emotional state (as in Study 2), and (d) the perceived levels of pain (as in Studies 1 and 2) while watching targets describing a negative social experience.

### Method

#### Participants

Forty-seven participants self-identified as British (39 women, *M*_age_ = 21.53 years) and 47 Chinese, all originating from Hong Kong, (34 women, *M*_age_ = 20.66 years) studying at a British University participated in a study on interpersonal relationships in exchange for £4. In the Chinese sample, 19.1% of the participants reported residing in the United Kingdom for less than 6 months, 27.7% for up to a year, 6.4% between 1 and 2 years, 17.0% between 2 and 5 years, 25.5% between 5 and 10 years, and 4.3% for more than 10 years. Again using the same scale as in Study 1, on average, East Asian participants self-rated that they were *average* to *somewhat fluent* in English (*M* = 3.81, *SD* = .88). The two samples were comparable in age, *t*(92) = .1.31, *p* = .19, thus age was not explored any further.

#### Stimulus development

The protocol outlined in Study 2 was used in the current study to generate videos of Chinese targets. Six Chinese female targets were invited to the lab and were videotaped describing in Cantonese two socially negative events they had experienced in their past. They received £4 for this task. After the completion of each recording, targets rated the affective valence, intensity and their own feelings as they described the event using the PANAS (see Study 2 for more details). Video targets granted permission to the researchers to use the recorded videos for future research.

Following the same criteria outlined in Study 2 used to determine video selection, we first short-listed the videos rated as most intense on the affect valence (less than 3 on the affect valence 9-point scale) and intensity scales (greater than 7 on the intensity 9-point scale). This screening resulted in 6 videos from a total of a pool of 12 videos. The final two videos were selected by the principal researchers based on content describing experiences likely to be common to all participants regardless of cultural background (i.e., being a victim of bullying and leaving friends behind).^7^

The two British target videos were the same as those used in Study 2. To address the potential language confound, the content in each video was translated by a bilingual speaker. Chinese subtitles were added to videos of British targets and English subtitles were added to videos of Chinese targets to aid nonnative speaker’s comprehension. A second independent bilingual speaker checked the translation for accuracy.

#### Main study procedure and measures

The study proceeded using the same protocol outlined in Study 2. Participants completed the study individually in the lab and were initially presented with an online questionnaire containing demographic questions. Next, they were presented in random order the two videos selected from the stimulus development phase in the current study (Chinese targets) and the two videos selected from the stimulus development phase in Study 2 (British targets). As participants watched the videos, they continuously indicated their own personal affective state using the affect rating dial used in the previous two studies. Participants then completed the PANAS items as did targets in the stimulus development phase, with instructions to judge the *target’s* feelings as the target was recalling the event in the video. Finally, participants completed a subset of emotional adjectives taken from the ERQ (Coke et al., 1978) to indicate their feelings of empathic concern. Once participants had watched all the videos and indicated their responses on all the measures, they were thanked, debriefed and paid for their participation.

##### Affect rating

As in Studies 1 and 2, affect rating was measured continuously during each video presentation using a rating dial (see Study 1 for details).

##### Empathic concern

The ERQ was used to assess feelings of empathic concern participants experienced while watching each of the videos (target_CH1_*:* α_BR_ = .88, α_CH_ = .79; target_CH2_; α_BR_ = .92, α_CH_ = .76; target_BR1_: α_BR_ = .83, α_CH_ = .76; target_BR2_: α_BR_ *= .*86, α_CH_ = .69) (see Study 2 for details). The same items used in Study 2 were used in the present study.

##### Perceived pain

Perceived pain scores were obtained using the same measure used in Studies 1 and 2.

##### Empathic accuracy

The same procedure described in Study 2 was used to compute absolute difference scores between each PANAS emotion score reported by the targets in the videos and those reported by the participants. All emotions were then collapsed to produce empathic accuracy scores in response to each target (target_CH1_*:* α_BR_ = .84, α_CH_ = .84; target_CH2_: α_BR_ = .80, α_CH_ = .82; target_BR1_: α_BR_ = .83, α_CH_ = .84; target_BR2_: α_BR_ = .86, α_CH_ = .76). As in Study 2, each average empathic accuracy scores was multiplied by −1 so that a lower score reflected lower empathic accuracy and a higher score reflected greater empathic accuracy.

### Results

As in the previous studies, we first examined whether the two cultural groups perceived comparable levels of pain in video targets. Next, we analyzed cultural differences in the outcome measures in response to observing social pain videos (see Table 3 for descriptive statistics). To this goal, we conducted separate 2 × 4 repeated-measures ANOVAs with perceived pain, affect rating, empathic concern, and empathic accuracy as dependent variables. In each ANOVA, cultural group (British vs. Chinese) was entered as the between-subjects variable and video target (video target_BR1_ vs. video target_BR2_ vs. video target_CH1_ vs. video target_CH2_) was entered as the within-subjects variable. Preliminary analyses that included sex as an additional factor revealed no significant main effects or interactions with sex; therefore, this variable was not included in the analyses reported below.[Table-anchor tbl3]

#### Perceived pain

The analysis revealed a significant main effect of video target, *F*(3, 276) = 60.34, *p* = .001, η_p_^2^ = .40. Participants perceived significantly more pain in video target_BR1_ (*M* = 4.59, *SD* = .89) and video target_BR2_ (*M* = 4.56, *SD* = .76) compared with video target_CH1_ (*M* = 4.27, *SD* = .75) and video target_CH2_ (*M* = 3.27, *SD* = .95) (all *p*s < .004)_._ There was no significant difference in perceived pain between the two British video targets (*p* = .83); however, more pain was perceived in video target_CH1_ compared to video target_CH2_ (*p* = .001)_._ In addition, there was a significant main effect of cultural group, *F*(1, 92) = 13.83, *p* = .001, η_p_^2^ = .13. Chinese participants (*M* = 4.35, *SD* = .51) perceived more pain compared with British participants (*M* = 3.99, *SD* = .43), *d* = .76. However, there was no significant cultural Group × Video target interaction, *F*(3, 276) = 1.75, *p* = .16, η_p_^2^ = .02. Given the possibility that any cultural differences found in empathic outcomes could potentially be attributed to cultural differences in perceived pain, and given the differences in perceived pain between targets, perceived pain score was added as a covariate in the analyses reported below. Finally, as in Study 2, we added video target as an additional factor in the analyses below as perceived pain differed significantly between video targets.

#### Affect rating

As in Study 2, the same data from the affect rating time series was extracted before any analyses and an identical process in data treatment was conducted to yield proportional affect rating scores. A 2 × 2 × 4 repeated-measures ANCOVA with the logged ratios of adjusted proportional affect rating scores (positive affect and negative affect) to the adjusted proportional no affect rating scores were entered as dependent variables. Cultural group (British vs. Chinese) was entered as the between-subjects variable, and both affective valence (positive vs. negative) and video target (video target_BR1_ vs. video target_BR2_ vs. video target_CH1_ vs. video target_CH2_) were entered as within-subjects variables. Similar to Study 2, we present the proportional affect rating scores to make interpretation easier (see Table 3).

This analysis revealed that the main effects of cultural group, *F*(1, 88) = 2.20, *p* = .14, η_p_^2^ = .02, affective valence, *F*(1, 88) = .98, *p* = .33, η_p_^2^ = .01, and video target, *F*(3, 264) = 1.96, *p* = .12, η_p_^2^ = .02) were not significant. The video Target × Affective valence, *F*(3, 264) = .86, *p* = .46, η_p_^2^ = .01, and the cultural Group × Affective valence interactions, *F*(1, 88) = .83, *p* = .36, η_p_^2^ = .01, were also not significant. However, there was a significant cultural Group × Video target interaction, *F*(3, 263) = 2.69, *p* = .05, η_p_^2^ = .03. Focusing on the cultural differences for each video target, the simple main effects revealed cultural differences in proportional affect in response to video target_CH1_, *F*(1, 88) = 5.00, *p* = .03, η_p_^2^ = .05, and marginally significant cultural differences in proportional affect in response to video target_CH2_, *F*(1, 88) = 3.34, *p* = .07, η_p_^2^ = .04. In response to each of the Chinese video targets, British participants reported proportionally more affect (video target_CH1_: *M* = −3.77, *SE* = .07; video target_CH2_: *M* = −3.59, *SE* = .09) compared with Chinese participants (video target_CH1_: *M* = −4.01, *SE* = .07; video target_CH2_: *M* = −3.83, *SE* = .07). There were no cultural differences in proportional affect in response to the British video targets: video target_BR1_, *F*(1, 88) = .11, *p* = .74, η_p_^2^ = .001, video target_BR2_, *F*(1, 88) = .45, *p* = .50, η_p_^2^ = .005. Finally, a significant three-way interaction emerged, *F*(3, 264) = 3.04, *p* = .03, η_p_^2^ = .03. Unpacking the three-way interaction and focusing on the cultural differences, we found cultural differences in proportional positive affect in response to both video target_CH1,_ *F*(1, 88) = 6.88, *p* = .01, η_p_^2^ = .07, and video target_CH2,_ *F*(1, 88) = 4.32, *p* = .04, η_p_^2^ = .05_._ In response to both Chinese video targets, British participants reported proportionally more positive affect (video target_CH1_: *M* = −3.88, *SE* = .08; video target_CH2_: *M* = −3.60, *SE* = .09) compared with Chinese participants (video target_CH1_: *M* = −4.20, *SE* = .08; video target_CH2_: *M* = −3.88, *SE* = .09). We should add that the direction of these findings did not change when mean scores of proportional affect were collapsed across the video targets in each cultural group.

#### Empathic concern

The ANCOVA with empathic concern revealed a significant main effect of cultural group, *F*(1, 88) = 16.90, *p* = .001, η_p_^2^ = .16. British participants reported more empathic concern for video targets (*M* = 2.02, *SE* = .13) compared with Chinese participants (*M* = 1.21, *SE* = .13), *d* = .69. The main effect of video target, *F*(3, 264) = 1.05, *p* = .37, η_p_^2^ = .01, and the cultural Group × Video target interaction, *F*(3, 264) = .89, *p* = .45, η_p_^2^ *= .*01, were not significant_._

#### Empathic accuracy

The ANCOVA with empathic accuracy revealed a significant main effect of cultural group, *F*(1, 88) = 3.82, *p* = .05. Chinese participants were more empathically accurate (*M* = −1.09, *SE* = .08) compared to British participants (*M* = −1.34, *SE* = .08), *d* = .49. There was a significant main effect of video target, *F*(3, 264) = 3.18, *p* = .02, η_p_^2^ = .04. Participants were significantly less accurate in inferring the emotions in video target_BR2_ (*M* = −1.52, *SE* = .06) compared to all other video targets (video target_BR1_: *M* = −1.20, *SE* = .06; video target_CH1_: *M* = −1.16, *SE* = .06; video target_CH2_: *M* = −1.02, *SE* = .06), all *p*s = .001_._ In addition, participants were significantly more accurate in inferring the emotions in video target_CH2_ compared to video target_BR1_ and video target_CH1_ (all *p*s = .001)_._ Finally, there was also a significant cultural Group × Video target interaction, *F*(3, 264) = 3.51, *p* = .02, η_p_^2^ = .04. Unpacking the interaction and focusing on the cultural differences for each video target, we found significant cultural differences in empathic accuracy had emerged for video target_BR1_, *F*(1, 88) = 4.04, *p* = .05, η_p_^2^ = .04, and video target_CH2,_ *F*(1, 88) = 9.50, *p* = .003, η_p_^2^ = .10. For both video targets, Chinese participants (video target_BR1_: *M* = −1.06, *SE* = .09; video target_CH2_: *M* = −.83, *SE* = .09) reported greater empathic accuracy compared to British participants (video target_BR1_: *M* = −1.34, *SE* = .09; video target_CH2_: *M* = −1.22, *SE* = .09). There were no cultural differences for the remaining video targets (all *p*s > 25).

Again, the reported significant cultural differences in empathic accuracy and empathic concern remained when mean scores of each outcome variable were collapsed across the video targets in each cultural group.

### Discussion

This study replicated two major findings observed in Study 2. First, British participants reported more empathic concern for the targets experiencing social pain than did Chinese participants. Second, Chinese participants were more empathically accurate than British participants. Of interest to the authors, Chinese participants reported greater empathic accuracy for a Chinese video target and a British video target suggesting that the cultural differences in empathic accuracy cannot be explained by an in-group advantage effect. The findings concerning proportional affect ratings to some extent follow the findings reported in Studies 1 and 2 where we found that British participants reported higher negative affect compared with East Asian participants, in response to physical pain (Study 1), and a greater proportion of affect in response to social pain (Study 2). In this study, we found that British participants reported greater proportional positive affect in response to Chinese video targets’ social pain only. As with the cultural differences in empathic accuracy, this cultural difference in proportional affect cannot be explained by an in-group advantage effect as British participants reported greater proportional affect for the targets from the outgroup. Similarly, although the difference was not significant, Chinese participants reported greater proportional affect for British video targets compared with British participants. Thus, the current findings suggest that, in real-time, participants did not empathize more with the targets from their in-group.

## General Discussion

In three studies designed to explore the role of cultural background in empathy, we demonstrated that cultural background is a meaningful moderator and plays a role in shaping both affective and cognitive empathic responses. Across two studies, we found that, compared with East Asian participants, British participants reported more negative affect/proportional negative affect as a response to observing physical (Study 1) and social pain (Study 2). Moreover, in two studies we found that, compared with East Asian participants, British participants reported more empathic concern but less empathic accuracy in response to observing someone suffering social pain (Studies 2 and 3).

### Implications for Culture and Affective Empathy

The current findings follow some of the patterns of cultural differences in different indices of empathy previously reported in the literature, but not others. On the one hand, the direction of the cultural difference in empathic concern observed in Studies 2 and 3 is in line with the heightened trait level empathic concern among Westerners that Cassels and colleagues (2010) measured using the empathic concern subscale of the IRI. On the other hand, the affect rating responses observed in both Studies 1 and 2, but not in Study 3, do not follow findings from Trommsdorff et al. (2007) and Cassels et al. (2010), who found greater personal distress reported by individuals of East Asian origin compared to individuals of Western origin. It should be noted that we measured affect rating in real-time while watching a physically or socially painful event, whereas previous studies have either examined personal distress cross-culturally at the trait level (Cassels et al., 2010) or coded distress responses using observational methods (Trommsdorff et al., 2007). Our approach to assessing affect provides a more detailed measurement that is likely to capture the online subjective emotional experience as the painful event evolves. This difference in methodology is one potential likely to account for the differences observed between current and past findings. More important, if emotional expression contributes to one’s well-being in Western cultures, as suggested by Soto et al. (2011) who demonstrated that emotional suppression is associated with greater depression and lower life satisfaction in this group, then the greater proportional affect and empathic concern evident among British participants in the current studies may be adaptive to their psychological functioning.

A second difference in methodology that could potentially account for the divergent results between the current and past findings concerns the distinction between personal distress and negative affect. Situations that evoke emotions of personal distress, in contrast to those that evoke empathic concern for example, yield significantly higher arousal, higher self-orientation, and more importantly, greater negative affect (Lopez-Perez, Carrera, Ambrona, & Oceja, 2014). Thus, there is a clear indication that negative affect is inherent in personal distress (Batson et al., 1987) and that negative affect, as measured by the rating dial in the current studies, reflects similar emotional processes encompassed in personal distress. Nevertheless, it is possible that other negative emotions (e.g., anger, frustration) might have been elicited in response to the video stimuli used in our studies. In fact, one could claim that negative affect, as measured in the current study, could reflect an other-oriented negative emotional response to the target. However, we think that this is unlikely given that the naturalistic stimuli videos used in the present studies were derived from common procedures designed to elicit an empathic response (e.g., Ma-Kellams & Blascovich, 2012) as opposed to an other-oriented negative response such as anger.

A further point to note about negative affect concerns an individual’s attitude for the affective state itself. For example, Koopmann-Holm and Tsai (2014) argued that an individual’s attitude toward negative affect may shape how they would respond to another’s suffering. Specifically, they showed that attitudes toward negative affect mediate cultural differences in the discomfort (or comfort) felt in focusing on the negative (vs. positive) aspects when expressing sympathy for a suffering individual. Therefore, it is possible that one’s attitude to a felt negative affective state might not be interpreted as personal distress, and that the interpretation of personal distress could differ as a function of one’s cultural background. We would like to note that these possible interpretations of findings on the affect rating should be taken with caution as the discrepancy between our findings and those reported in the literature were observed in Studies 1 and 2, but not in Study 3.

### Implications for Culture and Cognitive Empathy

Previous research has shown that compared with European Americans, East Asians exhibit a positive association between emotional suppression and interpersonal harmony (Wei et al., 2013) and a tendency to suppress both positive and negative emotions to maintain interpersonal harmony (Chiang, 2012). In addition, an accurate understanding of another’s emotional state is likely to assist interpersonal harmony maintenance. As Easterners (compared with Westerners) emphasize greater importance in maintaining interpersonal harmony (e.g., Ohbuchi et al., 1999), values of interpersonal harmony may have accounted for the dampened levels of affective empathy in our East Asian sample: both the negative affect reported in Studies 1 and 2 and the proportional positive affect reported in Studies 2 and 3. Moreover, values of interpersonal harmony may also account for the heightened levels of empathic accuracy in the East Asian sample compared with our British sample. It should be noted that the explanatory role of emotional suppression and values of interpersonal harmony were not assessed in the current studies, therefore, any interpretation of the current findings following this reasoning should be considered speculative and requires further research.

The current findings also do not follow Ma-Kellams and Blascovich’s (2012) findings that demonstrated greater empathic accuracy for strangers among Westerners, and greater empathic accuracy for close others among Easterners, relative to their cultural counterparts. The targets in our studies were strangers to participants, thus following Ma-Kellams and Blascovich’s (2012) reasoning, one could have expected the British participants in our studies to be more empathically accurate, which we did not find. However, although targets were strangers, both targets and participants were university students making them share an identity, which might have blurred the lines between in-group and out-group membership and this way closed the social gap between the targets and participants. Participants noticing these shared features may have perceived the targets less as strangers and “connected” with them (i.e., become closer to the targets). This possibility could also account for the lack of an in-group advantage in Study 3. Although cultural background is one variable that participants could use to distinguish in-group/out-group membership, other variables such as university student status, could shape perceived group membership identification. Future research should make both the distinction between in-group and out-groups more salient to participants, while controlling for familiarity to explain the discrepancy between the two sets of findings.

### No Evidence for In-Group Advantage Effect

In Study 3, we investigated whether a possible in-group advantage effect could explain the findings reported in Study 2. Specifically we tested whether the greater empathic concern and affect rating reported by British participants might have been because of targets and observers sharing the same ethnic group membership. However, no in-group advantage was found in Study 3 in any empathic outcome in either cultural group. Chinese participants were more empathically accurate regardless of the ethnicity of the target. On the same note, British participants were more empathically concerned regardless of the target ethnicity. Thus, the cultural differences in empathy observed in the current studies cannot be explained by an in-group advantage. In fact, the direction of findings from Study 3 suggests that an out-group advantage is present in both affective and cognitive empathic outcomes for both cultural groups.

### Limitations and Future Directions

Taken together, the current findings across the three studies suggest that different “strategies” to empathize (affective vs. cognitive) might be used as a function of one’s cultural background with British participants opting for a more affective empathic strategy and East Asian participants opting for a more cognitive strategy. The current findings imply that Westerners might place greater importance in feeling for another individual and Easterners might place greater importance in understanding the thoughts and feelings of another. Research demonstrates that the role of affective empathy and cognitive empathy can be dissociated from one another (e.g., Hynes, Baird, & Grafton, 2006; Shamay-Tsoory, Aharon-Peretz, & Perry, 2009), however, little is known about whether culture moderates the application of a particular empathic strategy. Future research should examine the extent of the moderating role of culture in empathic strategies and moreover, if one strategy over the other enables a greater cultural fit. Future research is also needed to examine if there is a cost associated with one strategy when opting for the alternative. One reason why British participants’ empathic accuracy was lower compared to East Asian participants might be because it was perhaps more important for them to acquire a feeling for the target, instead of understanding the thoughts and feelings of the target. Following the same reasoning, one reason why East Asian participants, compared to British participants, reported lower levels of affective empathy might be because it was perhaps more important for them to acquire an accurate understanding of the thoughts and feelings of a target, instead of feeling for the target.

It should be noted that the East Asian sample in all three studies consisted of a higher proportion of women compared to men. In general, the results showed limited sex effects in the studies presented. Future research should examine sex differences in more balanced samples with comparable number of women and men. It should also be noted that the demographic questions presented to participants at the beginning of each study, which contained questions referring to participants’ ethnicity, may imposed demand characteristics upon participants and subsequently influenced responses. However, the reported lack of an in-group advantage effect suggests that any questions referring to cultural background presented to participants in the beginning of each study is unlikely to have influenced participants’ responses. In addition, it could also be speculated about whether Hong Kong Chinese in Britain are the best cultural representatives of East Asia. However, we think that testing Hong Kong Chinese (as opposed to mainland Chinese) and British cultures renders a more conservative test. We still observe cultural differences between participants form a location once controlled by British and White British participants studying in a British university. This, in our view, provides a more stringent test and suggests that testing more prototypical representatives of the East Asian culture would likely show stronger effects.

A further point that we did not address in the current research and one that requires attention in future research is the potential behavioral consequences of the observed cultural differences. There is limited amount of culture comparative research examining the association between affective (e.g., empathic concern) and cognitive (e.g., empathic accuracy) empathic components on one hand and prosocial (or avoidant) behaviors on the other. For example, Trommsdorff et al. (2007) illustrates the relationship between empathic concern and prosocial behavior across cultures in preschoolers, replicating the general association between empathic concern and prosociality (e.g., Davis, 1983; Eisenberg & Miller, 1987; Mehrabian & Epstein, 1972). However, the association between cognitive empathy and prosociality across cultures has not been investigated using more varied interpersonal behavioral outcomes (e.g., conflict resolution).

In summary, in the present research we have found cultural differences in empathic responses to physical and social stimuli at both a cognitive and an affective level. Specifically, in contrast to East Asian participants, British participants reported greater negative affect in response to both physical and social pain, greater positive affect in response to social pain, and also had increased empathic concern that was accompanied by less empathic accuracy. These studies are the first to investigate cultural aspects of empathy to both social and physical pain while also distinguishing different aspects of empathy (personal distress, positive affect, empathic concern, and empathic accuracy) and this way they contribute to the sparse literature on the link between culture and empathy. The current findings demonstrate the importance of considering cultural background as a meaningful moderator of empathic responses and with an ever-shrinking world, one that warrants much greater attention in the future.

## Figures and Tables

**Table 1 tbl1:** Mean (SD) Scores for Affect Rating, and Perceived Pain Responses by Condition and Cultural Group (Study 1)

Condition	Affect rating^a^		Perceived pain^b^	
	British	East Asian	British	East Asian
	*M* (*SD*)	*M* (*SD*)	*M* (*SD*)	*M* (*SD*)
Hand-needle	3.10 (1.05)	4.00 (1.13)	3.95 (1.36)	3.70 (1.38)
Hand-Q-tip	5.18 (.67)	5.09 (.59)	1.21 (.41)	1.15 (.51)
Tomato-needle	4.47 (1.02)	4.07 (.93)	2.47 (1.52)	2.39 (1.35)
Tomato-Q-tip	5.35 (.72)	5.36 (.82)	1.05 (.32)	1.18 (.64)
^a^ 1 = *very negative* to 9 = *very positive.* ^b^ 1 = *no pain* to 6 = *hurts worst.*				

**Table 2 tbl2:** Mean (SD) Scores for Proportionate Affect Rating, Perceived Pain, Empathic Concern, and Empathic Accuracy Responses Separately for British and East Asian Cultural Samples (Study 2)

Measure	British cultural group		East Asian cultural group	
	Target 1	Target 2	Target 1	Target 2
	*M* (*SD*)	*M* (*SD*)	*M* (*SD*)	*M* (*SD*)
Proportion negative affect (%)	1.57 (1.20)	2.63 (1.66)	1.05 (.87)	1.85 (1.19)
Proportion positive affect (%)	1.39 (1.16)	1.65 (1.46)	.90 (.89)	1.04 (1.05)
Proportion no affect (%)	97.04 (2.35)	95.72 (3.05)	98.05 (1.75)	97.12 (2.18)
Perceived pain^a^	4.73 (.78)	4.44 (.69)	4.71 (.78)	4.56 (.87)
Empathic concern^b^	1.81 (1.16)	1.89 (1.01)	1.39 (.86)	1.12 (.64)
Empathic accuracy^c^	−1.18 (.33)	−1.28 (.44)	−1.03 (.33)	−1.17 (.30)
^a^ 1 = *no pain* to 6 = *hurts worst.* ^b^ 1 = *very slightly or not at all* to 5 = *extremely.* ^c^ −4 = *low empathic accuracy* to 0 = *high empathic accuracy.*				

**Table 3 tbl3:** Mean (SD) Scores for Proportional Affect Rating, Empathic Concern, Perceived Pain, and Empathic Accuracy Responses for Each Cultural Group in Response to British and Chinese Video Targets (Study 3)

Measure	British cultural group				Chinese cultural group			
	Target_BR1_	Target_BR2_	Target_CH1_	Target_CH2_	Target_BR1_	Target_BR2_	Target_CH1_	Target_CH2_
	*M* (*SD*)	*M* (*SD*)	*M* (*SD*)	*M* (*SD*)	*M* (*SD*)	*M* (*SD*)	*M* (*SD*)	*M* (*SD*)
Proportion negative affect (%)	1.33 (.88)	1.96 (1.16)	1.56 (1.03)	1.95 (1.61)	1.45 (1.46)	2.12 (2.27)	1.60 (1.21)	1.76 (1.77)
Proportion positive affect (%)	.71 (.90)	1.03 (1.16)	1.15 (1.18)	1.91 (1.44)	.85 (1.28)	1.08 (1.80)	.87 (1.25)	1.54 (1.81)
Proportion no affect (%)	97.96 (1.73)	97.01 (2.26)	97.29 (2.08)	96.14 (2.99)	97.70 (2.71)	96.80 (4.03)	97.53 (2.31)	96.71 (3.54)
Perceived pain^a^	4.53 (.86)	4.32 (.70)	4.13 (.68)	2.98 (.97)	4.64 (.92)	4.81 (.74)	4.40 (.80)	3.55 (.86)
Empathic concern^b^	1.95 (1.04)	2.04 (1.09)	1.76 (1.13)	1.93 (1.25)	1.15 (.86)	1.34 (.83)	1.22 (.91)	1.50 (1.04)
Empathic accuracy^c^	−1.31 (.60)	−1.54 (.68)	−1.15 (.61)	−1.57 (1.20)	−1.09 (.58)	−1.51 (.55)	−1.17 (.61)	−.85 (.51)
^a^ 1 = *no pain* to 6 = *hurts worst.* ^b^ 1 = *very slightly or not at all* to 5 = *extremely.* ^c^ −4 = *low empathic accuracy* to 0 = *high empathic accuracy.*								

## Footnotes

1 There was a significant cultural Group × Sex interaction with perceived pain as the dependent variable, *F* (1, 67) = 4.05, *p* = .05, η_p_^2^ = .05. However, the simple main effects revealed neither significant cultural differences for each sex (all *p*s > .14), nor sex differences for each cultural group (all *p*s > .13).

2 Although the main effect of video target was not significant, *F* (3, 195) = .29, *p* = .84, η_p_^2^ = .004, both the main effect of cultural group, *F* (1, 65) = 8.58, *p* = .01, η_p_^2^ = .12, and the cultural Group × Condition interaction, *F* (3, 195) = 4.401, *p* = .01, η_p_^2^ = .06, remained significant when controlling for perceived pain. The direction of the findings were as reported in the affect rating results.

3 Intensity and affect valence ratings for video target 1 equaled 9 and 1, respectively. For video target 2, intensity and affect valence ratings equaled 8 and 1, respectively.

4 Of interest to the authors, controlling for both perceived pain for each video target eliminated both the main effects of affective valence, video target, and the video Target × Affective valence interaction. More important, the main effect of cultural group remained and revealed the same pattern of results reported in the main analysis, *F* (1, 83) = 8.55, *p* = .004, η_p_^2^ = .09. In addition, there was a significant cultural Group × Sex interaction with the logged ratios of adjusted proportional affect rating scores (positive affect and negative affect) to the adjusted proportional no affect rating scores as dependent variables, *F* (1, 82) = 4.94, *p* = .03, η_p_^2^ = .06. The simple main effects revealed that British female participants (*M* = −3.60, *SD* = .46) reported significantly greater proportional affect compared to East Asian female participants (*M* = −3.96, *SD* = .36), *F* (1, 82) = 11.26, *p* = .001, η_p_^2^ = .12. In addition, East Asian male participants (*M* = −3.67, *SD* = .39) reported significantly greater proportional affect compared to East Asian female participants, *F* (1, 82) = 4.04, *p* = .05, η_p_^2^ = .05. There were no significant cultural differences in the male sample in proportional affect, *F* (1, 82) = .13, *p* = .72, η_p_^2^ = .002, and no sex differences in the British sample in proportional affect, *F* (1, 82) = 1.09, *p* = .30, η_p_^2^ = .01.

5 Both the main effect of cultural group, *F* (1, 82) = 10.98, *p* = .001, η_p_^2^ = .12, and the cultural Group × Video target interaction, *F* (1, 82) = 4.29, *p* = .04, η_p_^2^ = .05, remained significant when controlling for perceived pain. The direction of the findings were as reported in the affect rating results.

6 Although the main effect of video target was not significant, *F* (1, 82) = 2.67, *p* = .11, η_p_^2^ = .31, the main effect of cultural group, *F* (1, 82) = 4.33, *p* = .04, η_p_^2^ = .05, remained significant when controlling for perceived pain. The direction of the findings did not change as those reported in the results of Study 2.

7 Intensity and affect valence ratings for Chinese video target 1 equaled 7 and 3, respectively. For Chinese video target 2, intensity and affect valence ratings equaled 8 and 1, respectively.
